# Community-based interventions that work to reduce HIV stigma and discrimination: results of an evaluation study in Thailand

**DOI:** 10.7448/IAS.16.3.18711

**Published:** 2013-11-13

**Authors:** Aparna Jain, Ratana Nuankaew, Nungruthai Mongkholwiboolphol, Arunee Banpabuth, Rachada Tuvinun, Pakprim Oranop na Ayuthaya, Kerry Richter

**Affiliations:** 1 International Center for Research on Women,Washington, DC, USA; 2 Population & Community Development Association (PDA), Bangkok, Thailand; 3 Pact Thailand/Greater Mekong Region, Bangkok, Thailand; 4 Institute for Population and Social Research, Mahidol University, Salaya, Thailand

**Keywords:** evaluation, HIV, AIDS, stigma, discrimination, Thailand, PLHIV

## Abstract

**Introduction:**

HIV stigma and discrimination are major issues affecting people living with HIV in their everyday lives. In Thailand, a project was implemented to address HIV stigma and discrimination within communities with four activities: (1) monthly banking days; (2) HIV campaigns; (3) information, education and communication (IEC) materials and (4) “Funfairs.” This study evaluates the effect of project interventions on reducing community-level HIV stigma.

**Methods:**

A repeated cross-sectional design was developed to measure changes in HIV knowledge and HIV-related stigma domains among community members exposed to the project. Two cross-sectional surveys were implemented at baseline (respondent *n*=560) and endline (respondent *n*=560). *T*-tests were employed to assess changes on three stigma domains: fear of HIV infection through daily activity, shame associated with having HIV and blame towards people with HIV. Baseline scales were confirmed at endline, and each scale was regressed on demographic characteristics, HIV knowledge and exposure to intervention activities.

**Results:**

No differences were observed in respondent characteristics at baseline and endline. Significant changes were observed in HIV transmission knowledge, fear of HIV infection and shame associated with having HIV from baseline to endline. Respondents exposed to three specific activities (monthly campaign, Funfair and IEC materials) were less likely to exhibit stigma along the dimensions of fear (3.8 points lower on average compared to respondents exposed to none or only one intervention; 95% CI: −7.3 to −0.3) and shame (4.1 points lower; 95% CI: −7.7 to −0.6), net of demographic controls and baseline levels of stigma. Personally knowing someone with HIV was associated with low fear and shame, and females were less likely to possess attitudes of shame compared to males.

**Conclusions:**

The multivariate linear models suggest that a combination of three interventions was critical in shifting community-level stigma – monthly campaign, Funfair and IEC materials. This is especially important given Thailand's new national AIDS strategy to reduce HIV-related stigma and discrimination by half by 2016. Knowing which interventions to invest in for HIV stigma reduction is crucial for country-wide expansion and scale-up of intervention activities.

## Introduction

In Thailand, the first reported case of HIV dates back to 1984 [1]. Since that time, the primary mode of transmission has been unprotected sex [2]. In the early 1990s, prevention and control of HIV infections became national priorities, and mass communication campaigns were implemented to increase awareness about HIV and AIDS. Prevalence rates had exceeded 30% among female sex workers by 1995 [3], and the “100% Condom Campaign” was implemented to encourage condom use among all female sex workers and their clients [4]. Widespread condom distribution was instituted throughout the country. Significant progress has been made to curb the spread of HIV; in 2009, the prevalence rate was at 1.3%, with roughly 530,000 living with HIV and an estimated 12,000 new infections [5].

Despite this progress, HIV stigma and discrimination are major issues affecting people living with HIV (PLHIV) in their everyday lives. Worldwide, HIV-related stigma and discrimination are recognized as facilitators in the spread of HIV infections, barriers in the practice of safe and effective HIV-prevention behaviours and significant obstacles in the access of HIV care, treatment and support services [6–9]. HIV stigma and discrimination are the disapproval or devaluation of PLHIV where members of society set PLHIV apart from ordinary activities. In an earlier study conducted in Thailand, researchers found that community members believed that PLHIV should not participate in community activities and should be restricted to their homes [10]. Research in Thailand and Vietnam has shown that the consequences of HIV stigma are severe and may lead to loss of livelihood, refusal of care and depression [11–13]. A 2009 study found that Thai PLHIV have high levels of self-stigma, and that they suffer job loss and refusal of healthcare services, including family planning services [14].

In this study, a repeated cross-sectional design was used to measure increases in HIV knowledge and reductions in HIV-related stigma domains among community members who were exposed to a community-based economic development project. Besides exploring the effect of the project activities on changes in knowledge and stigma, attention is paid to identifying the specific activities that appear to be responsible for the changes.

## Project overview

Launched in 2002, the Population and Community Development Association (PDA) implemented the Positive Partnership Project (PPP), which was designed to economically empower PLHIV, increase their quality of life and reduce the stigma and discrimination they encounter. To economically empower and increase the quality of life of PLHIV, the project provided low-interest loans to a buddy pair consisting of a person living with HIV and an individual without HIV. PLHIV chose their buddy, who was often someone they already knew like friends or family members, and who they had already disclosed their status to. The pair received training to build their skills in marketing, accounting and business management to ensure the success of their commercial endeavours. An additional objective focused on reducing HIV stigma and discrimination within the communities where the loan recipients lived [15]. The project was documented by UNAIDS as a “Best Practice” of PLHIV economic empowerment [16].

Phase II of the project began in April 2008, and two project models were developed and implemented to ensure sustainability beyond the project period: (1) PPP clubs that formed organically by PLHIV support groups; and (2) village development banks (VDBs) that formed mostly by community members and leaders. Both models were responsible for dispersing loans to the buddy pairs, collecting savings, and conducting HIV awareness-raising activities in their communities. Eleven PPP clubs and 12 VDBs were established in 23 communities across six Thai provinces: Bangkok, Chiang Mai, Chiang Rai Chonburi, Khon Kaen and Nakhon Ratchasima.

From September 2009 to September 2010, the project implemented specific HIV-stigma reduction interventions in the communities, including (1) monthly banking days; (2) HIV campaigns; (3) information, education and communications (IEC) materials; and (4) “Funfair” events. The monthly banking days were an important mechanism to continuously mobilize and unite the community. During these days, financial activities for the buddy pairs and other VDB/PPP club members were undertaken (e.g., deposits and loan repayments). In addition, HIV education activities were conducted, including, for example, inviting a PLHIV who was open about his or her status to share experiences with HIV stigma and discrimination. The PPP club and VDB members (consisting of both individuals living with HIV and those without HIV) developed HIV campaigns that were disseminated in their communities. These campaigns were conducted continuously throughout the project period and included activities such as condom distribution as well as household visits to share HIV information, engage community members in discussions around HIV, provide community members opportunities to discuss concerns and doubts, and raise awareness of HIV stigma. The IEC materials were developed based on the baseline survey results and in close collaboration with PLHIV involved in the project. Three IEC materials were developed specifically under this project with key HIV stigma and discrimination messaging. The IEC materials included posters, banking slips with key messages and radio dramas. Examples of key messages include *Being infected with HIV and AIDS is not shameful* and *We should not blame PLHIV and think of them as promiscuous*. Finally, “Funfair” events were held every six months; these were a combination of education and entertainment activities, such as quizzes, role plays and exhibitions.

The paired buddies supported one another to repay their joint loan as well as participate in HIV and AIDS awareness-raising activities with PDA staff and VDB and PPP club committee members. The intervention emphasized contact strategies of working together and supporting one another on these activities to model productive and supportive interactions between individuals living with HIV and those without HIV. These interactions were intended to model positive relationships with PLHIV so that community members could overcome their fears around casual contact with PLHIV and reduce negative attitudes and stereotypes towards PLHIV. These activities were also intended to address the negative attitudes of buddies towards PLHIV and the internalized stigma among PLHIV.

## Methodology

### Data

This study uses community-level surveys that were part of a broader evaluation study aimed at assessing overall project activities, including increases in quality of life of PLHIV, and reductions in HIV stigma and discrimination among buddies. The broader evaluation study implemented surveys with PLHIV involved in the project, the buddies and the family members of project participants (PLHIV and buddies) [17]. The current study only uses data collected from community members. For the community-level survey, the same 11 communities were surveyed, and two cross-sectional surveys were implemented at baseline and endline. A sampling frame of households was developed in each community, and households were selected using systematic random sampling. All individuals 15 years and older were interviewed in each household. Data collection for the baseline and endline survey were conducted from October 2008 to March 2009 and from November 2010 to January 2011, respectively. An equal number of community respondents were interviewed at baseline (*n*=560) and endline (*n*=560).

### Ethical review

The Institutional Review Board at Mahidol University (Salaya, Thailand) reviewed and approved the baseline and endline study designs. Data collectors were trained in implementing the informed consent procedure, and verbal informed consent was obtained from all respondents at baseline and endline.

### Stigma measures

A series of previously validated and tested stigma measures [18, 19] and new items specific to the Thai context were used in the survey. The measures were developed to capture two drivers of stigma: fear of HIV infection and social judgement. Fear of infection items capture fear of HIV transmission in specific casual encounters (e.g., exposure to the saliva or sweat of a PLHIV, or sharing a meal with a PLHIV). The social judgement items include attitude questions related to blaming PLHIV for acquiring the disease and feelings of shame or disgrace associated with having HIV. Fear of infection associated with casual encounters plus social judgement might lead to damaging behaviours, such as avoidance, isolation or gossip.

### Dependent variables

We developed scales using principal component factor analysis to identify uni-dimensional constructs at baseline. The first scale captured fear-based stigma, while the second scale measured attitudes related to shame. These factors were confirmed at endline using confirmatory factor analysis. We tested the reliability of the scales using Cronbach's alpha with a cutoff of 0.7 [20]. Scale validity was assessed, and predicted regression scores were obtained. The scales were standardized to have a mean of 50 and a standard deviation of 10, and they ranged from 0 to 100 where higher scores indicated higher levels of stigma.

### Main predicator

The main predictor was respondents’ reported exposure to the interventions. Four interventions were assessed: (1) monthly meetings on banking days, (2) HIV campaign, (3) IEC materials with key messages (posters, messages on banking slips and radio dramas) and (4) Funfair events. Each intervention was assessed individually and as a dose–response relationship. The dose–response variable is an additive variable of exposure to one, two, three or all four activities. In the analysis, we also identified specific combinations of interventions to predict levels of stigma. We obtained intervention exposure from respondents with both unprompted and prompted questions to determine whether exposure was to PPP project activities or something else. We first asked, for example, “In the past 12 months, have you participated in any Positive Partner Project activities?” If yes, we asked respondents to describe the project activities that they have participated in. If no, we asked, “In the past 12 months, have you ever participated in the following activities?” and then we asked about each activity that was not spontaneously recalled. For the IEC materials, we used a similar process where we first asked respondents to recall specific messages spontaneously. Of the messages that were not recalled spontaneously, we asked them whether they remembered seeing and being exposed to specific messages. Additional predictors of interest included personally knowing someone living with HIV and HIV transmission knowledge.

### Covariates

Study covariates included gender, marital status, age, education, residence, personal income level, occupation and media exposure to HIV messaging. Gender is classified as male or female. A four-category age variable was constructed of 15–29, 30–39, 40–49 and 50+ years old. Marital status was grouped into married versus single, divorced or widowed, and residence was grouped into urban versus rural. Education is classified into no education or primary, and secondary or higher. Personal average monthly income was categorized into four groups: less than 3000 Baht (equivalent to less than $102), 3000–4999 Baht (approximately $102–170), 5000–6999 Baht (approximately $170–238) and 7000 Baht or more ($238 or more). Six occupational types were formed: farmers, small business owners, government employees, factory workers or casual labourers, students, and housewives or unemployed.

We included respondents’ exposure to non-project-related HIV and AIDS information in the past 12 months as another covariate. Four information sources were assessed: radio, television, newspapers and posters. Finally, we created two continuous baseline community-level variables to account for potential differences in community-level stigma before the interventions. We created an average score of fear at baseline in each community surveyed. Across all communities, the fear score ranged from 44.5 to 54.1. A similar variable was generated for baseline community-level shame. The score ranged from 43.0 to 57.0 across all communities.

### Statistical analysis

We compared baseline and endline respondent characteristics and assessed change from baseline to endline in items comprising the fear scale and the shame scale using Wald Chi-square analysis and a two-tailed significance level with a *p*<0.05. Bivariate linear regression analyses for each respondent characteristic and the outcome scales were conducted. Multivariate linear regression models were run of three main predictor variables on the fear and shame scales, adjusted for respondent characteristics and a baseline community average of stigma. All analyses are conducted in STATA.SE, Version 12 [21].

## Results

### Respondent profile

Table 1 presents frequency distributions of respondents’ background characteristics by survey round. A slightly larger proportion of females were interviewed at baseline and endline (58.6% and 58.8%), and an equal proportion of individuals were interviewed in rural and urban areas in both survey rounds. The majority of respondents at baseline and endline were married, were over the age of 40, had less than primary education and were employed as farmers, factory workers or casual labourers. A greater proportion of respondents made 7000 Baht ($238) or more per month at endline when compared to baseline (*p*=0.00). Aside from personal income, there were no statistically significant differences in respondent characteristics at baseline and endline.

**Table 1:** Frequency distributions of respondent characteristics by survey round

	Baseline (*n*=560)	Endline (*n*=560)
Gender		
Female	58.6	58.8
Male	41.4	41.2
Residence		
Urban	50.0	50.0
Rural	50.0	50.0
Marital status		
Married	79.5	75.4
Single, divorced or widowed	20.5	24.6
Age		
15–29	22.5	20.0
30–39	17.3	19.5
40–49	23.0	23.2
50 and above	37.2	37.3
Mean age	43.0	43.7
Median age	44.0	45.0
Education		
None or primary	58.1	55.5
Secondary, high school or vocational	33.9	36.6
University or BA	8.0	7.9
Occupation		
Farmer	23.9	20.7
Small business owner	17.5	17.0
Private or government employee	10.0	11.9
Factory worker or casual labourer	26.1	25.9
Student	5.5	6.8
No occupation, or housewife	17.0	17.7
Average monthly income		
<3000 Baht	37.9	35.6
3000–4999 Baht	21.6	14.6
5000–6999 Baht	18.0	15.2
≥7000 Baht	22.5	34.6

### HIV knowledge

**Figure 1:**
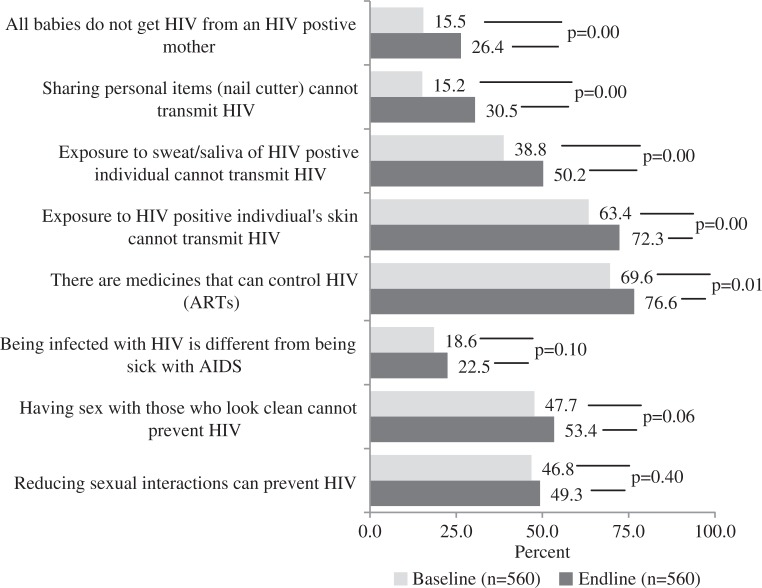
Change in frequency distributions on correct HIV knowledge at baseline and endline.

Respondents’ knowledge of HIV transmission, prevention and treatment was assessed at baseline and endline. Across nine questions, correct knowledge increased significantly on the first five questions presented in Figure 1. For example, more respondents at endline knew that all babies do not get HIV from a mother living with HIV and that exposure to the sweat or saliva of a person living with HIV cannot transmit HIV. Low levels of knowledge persisted at endline for the difference between HIV and AIDS at 22.5%. One HIV knowledge question did not change from baseline to endline: 77% of respondents reported that HIV and AIDS is only transmitted among people who inject drugs (PWID), female and male sex workers (F/MSW) and men who have sex with men (MSM) (data not shown).

### Fear of HIV infection and social judgement stigma

The stigma items measured for the fear scale were associated with fear of HIV transmission through casual encounters and everyday contact with PLHIV. The factor loadings and alphas of the fear scale from baseline to endline are presented in Table 2.

**Table 2:** Fear of HIV infection stigma scale: factor loadings and Cronbach's alpha

	Baseline	Endline
Being exposed to saliva of PLHIV	0.752	0.715
Being exposed to sweat of PLHIV	0.772	0.770
Having a meal or sharing food with PLHIV	0.710	0.723
Using the same plate, spoons or forks as PLHIV	0.732	0.733
Taking care of PLHIV	0.726	0.722
Carrying PLHIV	0.734	0.754

Cronbach's alpha	0.83	0.85

**Figure 2:**
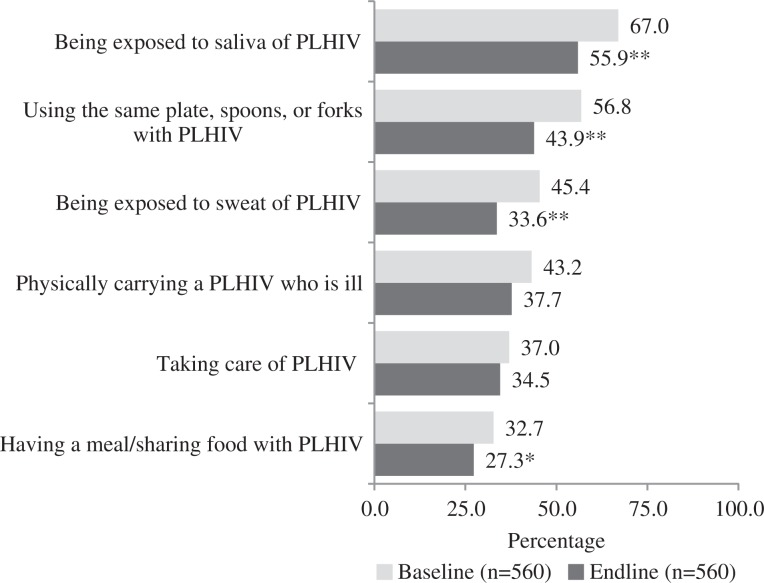
Change in percentage of fear in fear of HIV infection stigma items from baseline to endline. **p<0.01; *p<0.05.

Figure 2 presents the change in fear of HIV infection from baseline to endline on six measures. While reductions in fear were observed across all six stigma items, statistically significant declines were seen in four items: (1) exposure to the saliva of an individual living with HIV, (2) sharing cutlery with an individual living with HIV, (3) exposure to the sweat of an individual living with HIV and (4) having a meal (sharing food) with an individual living with HIV. When disaggregated by gender, similar patterns of significance were observed for females and males, except that physically *carrying a PLHIV who is ill* declined significantly among females but not males.

**Figure 3:**
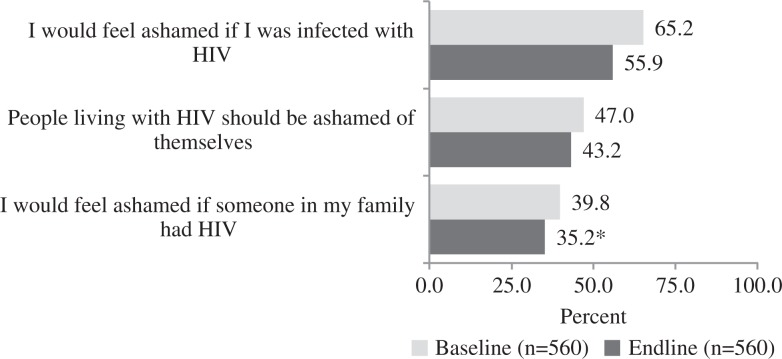
Change in percentage of agreement on social judgement stigma from baseline to endline. **p<0.01; *p<0.05.

Table 3 presents the factor loadings and alphas for the social judgement scales at baseline and endline. Figure 3 presents the percentage of agreement with shame scale items at baseline and endline. At baseline, 39.8% of respondents agreed with the statement that they would feel ashamed if someone in their family had HIV. This dropped slightly to 35.2% at endline (*p*<0.05). A non-statistically significant decline was observed in the other two social judgement stigma measures. The three shame scale items were disaggregated by gender, and results showed declines on all three items among females and males, but significance was observed only across the three items with females (data not shown). Two items were assessed to measure attitudes that blamed individuals for contracting HIV. The distribution frequencies of these two items did not change from baseline to endline (data not shown), and therefore a blame scale was not developed or assessed in the multivariate models.

**Table 3:** Social judgement stigma scales: factor loadings and Cronbach's alpha

	Baseline		Endline	
		
	Shame	Blame	Shame	Blame
PLHIV should be ashamed of themselves	**0.769**	−0.013	**0.701**	0.046
I would feel ashamed if someone in my family had HIV and AIDS	**0.841**	−0.010	**0.842**	−0.021
It is the promiscuous men who spread HIV in your community	0.006	**0.955**	−0.018	**0.950**
It is the promiscuous women who spread HIV in your community	0.005	**0.954**	0.025	**0.942**
I would feel ashamed if I was infected with HIV	**0.768**	0.046	**0.811**	0.002

Cronbach's alpha	0.71	0.90	0.69	0.88

The bold numbers indicate which factor the item loaded on.

### Intervention exposure

**Figure 4:**
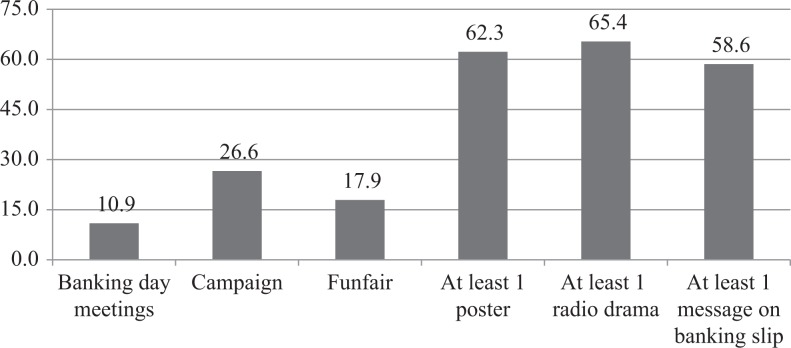
Frequency distributions of exposure to specific project interventions among endline respondents (*n*=560).

**Figure 5:**
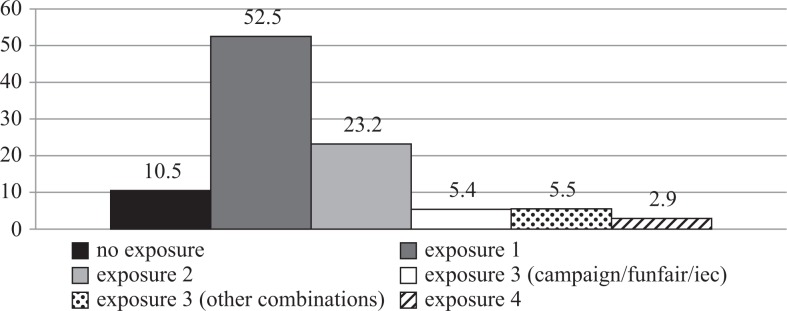
Frequency distributions of exposure to multiple project interventions among endline respondents (*n*=560).

Figures 4 and 5 show frequency distributions of exposure to project interventions. At endline, 10.9% of respondents reported participating in a banking day meeting, 26.6% reported exposure to the HIV campaigns and 17.9% reported participation in a Funfair event. In terms of IEC materials, over half of respondents reported exposure to at least one poster (62.3%), at least one radio drama (65.4%) and at least one message on a banking slip (58.6%; Figure 4). Only 1 in 10 respondents was not exposed to any project activities (10.5%; Figure 5). The majority of respondents were exposed to one activity (52.5%), while only a small proportion was exposed to all four activities (2.9%).

### Multivariate analyses

For both scales, we first ran unadjusted models with each intervention separately and found no significant associations. Then, we looked at the unadjusted dose–response relationship and found that exposure to three interventions was significant for both stigma scales (data not shown). We were interested in identifying the three interventions that yielded these results and learned that respondents who reported participation in or exposure to HIV campaign, Funfair, and IEC scored 5.21 points (95% CI: −8.86 to −1.54) lower on the fear scale and 5.14 points (95% CI: −8.80 to −1.49) lower on the shame scale (data not shown). Next, we adjusted the multivariate linear regression models for fear and shame stigma scales on project intervention exposure, HIV and AIDS knowledge and personal association with someone living with HIV, net of respondent's gender, marital status, age, education, residence, personal income, occupation, media exposure to non-project HIV messages and baseline community average of fear (Table 4). Table 4 shows the beta coefficients and 95% confidence intervals of the main predictors. Models I and II present the dose–response association of the number of project activities exposed to and reductions in fear of HIV infection stigma and social judgement stigma, respectively. The association between participation and exposure to HIV campaign, Funfair and IEC materials remains in the adjusted model for fear (3.81 points lower; 95% CI: −7.32 to −0.30) and shame (4.12 points lower; 95% CI: −7.67 to −0.58).

**Table 4:** Adjusted* multivariate linear regressions of fear of HIV infection stigma and social judgement stigma among endline respondents (*n*=560)

	Model I: fear of HIV infection (fear scale)		Model II: social judgement (shame scale)	
		
	*β*	(95% CI)	*β*	(95% CI)
Intervention exposures				
None or one	Ref		Ref	
Two	−1.13	(−3.12: 0.86)	−0.60	(−2.62: −1.43)
Three (campaign, Funfair and IEC)	−3.81	(−7.32: −0.30)	−4.12	(−7.67: −0.58)
Three (other intervention combinations)	−2.62	(−6.25: 1.02)	−1.24	(−4.93: 2.46)
Four	−0.93	(−5.73: 3.88)	−3.87	(−8.74: 1.01)
HIV and AIDS knowledge				
0–3 correct responses	Ref		Ref	
4–8 correct responses	−4.92	(−6.57: −3.27)	−3.76	(−5.42: −2.09)
Personally know someone living with HIV				
No	Ref		Ref	
Yes	−2.62	(−4.51: −0.72)	−1.89	(−3.80: 0.03)
Constant	66.46	(42.09: 90.84)	42.94	(28.86: 57.02)

* Models were adjusted for respondent's gender, marital status, age, education, residence, personal income, occupation, media exposure to HIV messages and baseline community average of fear.

In terms of gender, no significant differences were observed between females and males on the fear of infection scale. However, females are less likely to possess attitudes of shame compared to males (2.41 points lower; 95% CI: −4.14 to −0.68; data not shown).

The adjusted multivariate linear models also showed that when respondents personally know someone living with HIV, they are more likely to have lower scores on the fear of HIV infection stigma scale (2.62 points lower; 95% CI: −4.51 to −0.72). Higher HIV knowledge at endline predicted lower levels of fear and social judgement. Respondents with higher knowledge [i.e., those who answered 4–8 knowledge questions correctly (58.2%)] scored 4.83 points (95% CI: −6.48 to −3.19) lower on the fear scale and 3.79 points (95% CI:−5.46 to−2.12) lower on the social judgement scale than respondents with lower knowledge. Further analysis showed that the effect of personally knowing someone living with HIV on fear of HIV infection was significant only among those with low HIV knowledge, whereas the effect of high knowledge was significant, regardless of whether or not the respondent knows someone living with HIV (data not shown).

We explored the relationship of HIV knowledge as a potential mediator of the relationship between intervention exposure and fear of HIV infection and social judgement. The results of this analysis suggest that when HIV knowledge was included in the multivariate analysis, it attenuated the observed relationship between intervention exposure and fear and social judgement slightly (regression coefficient reduces from −5.21 to −4.89 for fear and from −5.14 to −4.91 for shame; data not shown).

## Discussion

The results of this study show that participation and exposure in project activities are associated with declines in fear of HIV infection and social judgement stigmas. The dose–response relationship assumes that different levels of exposure influence changes in fear of HIV infection and social judgement stigmas. While incremental changes in the outcome were not observed with each additional exposure, three interventions were identified as necessary for addressing fear of HIV infection and social judgement stigmas in Thailand. The three interventions – HIV campaigns, IEC materials and the Funfair – provide information about what actions and behaviours are stigmatizing, the consequences of stigma experienced by a person living with HIV, resources for treatment and care, and methods to prevent transmission, among other information. These three interventions communicate these types of information through various modes, including opportunities for community members to receive answers to questions and alleviate doubts (through the household visits and banking days), personal interactions with PLHIV (VDB and PPP clubs), anti-HIV stigma messaging reinforced through a wide array of communication modes (posters, banking slips and radio dramas) and hosted events that engaged the community in fun activities such as games and quizzes in addition to staging role-plays that addressed these issues (Funfairs). As documented elsewhere [20] and revealed in this study, by addressing stigma through a combination of activities, individuals were offered a variety of mechanisms and opportunities to be exposed to HIV stigma-reduction issues, which resulted in reductions in fear of HIV transmission and stigmatizing attitudes.

Our findings demonstrate that although community members’ knowledge of HIV transmission, prevention and treatment increased significantly on certain items, there is still considerable work to be done to improve knowledge levels. Roughly one in two individuals in our endline sample did not know that HIV cannot be transmitted through sweat or saliva, and one in four individuals did not know that HIV cannot be transmitted through skin contact. The study findings also show that increasing HIV knowledge may be an initial stage in addressing fear of HIV transmission and stigmatizing attitudes. The links between increase in HIV knowledge and decrease in fear, and increase in HIV knowledge and decrease in negative attitudes, have been demonstrated in Chiang Rai, Thailand [22] and Ethiopia [23]. Once individuals possess correct information about how HIV can and cannot be transmitted, fears of HIV infection in daily interactions with PLHIV can be diminished. Further analysis of knowledge as a potential mediator suggests that intervention exposure, in part, influences stigma through HIV knowledge but does not fully explain the effect of intervention activities on stigma.

The multivariate analysis revealed that individuals who know someone living with HIV have less fear of HIV transmission and less stigmatizing attitudes towards PLHIV. This has been shown in Thailand elsewhere [24, 25] and in several other studies [23, 26]. Researchers of a multi- country study found a negative correlation between HIV prevalence and negative attitudes towards PLHIV [27], and concluded that contact with PLHIV is more common in high-prevalence settings because HIV is normative, which might influence attitudes. While our study was in a low-prevalence setting, contact with PLHIV reduces stigma only among those who have low HIV knowledge. The effect of high knowledge on stigma, however, is significant, regardless of whether or not the respondent knows someone living with HIV, suggesting that knowledge has a greater influence on fear of HIV infection stigma than knowing someone living with HIV.

There are several limitations that need to be considered while interpreting the results. First, no control communities were included in the original design of the study. Therefore, we cannot claim that the interventions are responsible for the observed changes in HIV stigma. In our analysis, however, we did adjust for exposure to other HIV messaging that might have coincided with the intervention activities. Second, we were unable to account for correlation among individuals residing in the same household in this analysis. Also, the analysis does not reflect the level of participation in interventions or intensity of exposure. Finally, the results may also be subject to social desirability given that this topic is highly sensitive. The estimates on the fear and shame scales might be an underestimate if respondents are unwilling to share stigmatizing attitudes in one-on-one interviews.

## Conclusions

The purpose of this study was to determine changes in HIV knowledge and negative attitudes towards PLHIV among community members exposed to the PPP project. The results of this study suggest that programmes that choose to focus on HIV-related stigma reduction need to address the issue in multiple ways. Developing three interventions – Funfair, IEC materials and the HIV campaign (condom distribution and household visits) – led to a shift in knowledge and attitudes associated with fear of HIV and shame. Programmes also need to address multiple domains of stigma – knowledge, fear, shame and blame –simultaneously while recognizing that blame is a harder construct to reduce. Social judgement stigma is a harder construct to shift, as also shown in a Vietnam study [19] where interventions reduced fear and shame but the reductions in shame were smaller. Social judgement stigma tends to be deep rooted, and for future programming a longer intervention period may be needed. Also, specific interventions designed to tackle blame may be more effective. For example, programme might develop safe, nonthreatening spaces for honest and open discussion among individuals to better understand what drives these blaming attitudes.

This research is especially important given Thailand's new national AIDS strategy aligned with the UNAIDS vision of “getting to zero” – zero new HIV infections, zero discrimination and zero AIDS-related deaths. One of the goals of this new strategy is to reduce HIV-related stigma and discrimination by half by 2016 [28]. The findings of this study provide a good starting point for the programme to consider expanding to achieve this goal.
